# A Smartphone App to Restore Optimal Weight (SPAROW) in Women With Recent Gestational Diabetes Mellitus: Randomized Controlled Trial

**DOI:** 10.2196/22147

**Published:** 2021-03-16

**Authors:** Karen Lim, Shiao-Yng Chan, Su Lin Lim, Bee Choo Tai, Cammy Tsai, Su Ren Wong, Siew Min Ang, Tong Wei Yew, E Shyong Tai, Eu Leong Yong

**Affiliations:** 1 Department of Obstetrics and Gynecology, National University Hospital Yong Loo Lin School of Medicine National University of Singapore Singapore Singapore; 2 Department of Dietetics National University Hospital Singapore Singapore; 3 Saw Swee Hock School of Public Health National University of Singapore Singapore Singapore; 4 Department of Rehabilitation National University Hospital Singapore Singapore; 5 Department of Medicine National University Hospital Singapore Singapore; 6 Department of Medicine Yong Loo Lin School of Medicine Singapore Singapore

**Keywords:** randomized controlled trial, gestational diabetes mellitus, prevention, weight loss, mobile phone

## Abstract

**Background:**

Women with a history of gestational diabetes mellitus (GDM) are at an increased risk of developing type 2 diabetes mellitus (T2DM). Lifestyle interventions aimed at postpartum weight loss to reduce T2DM risk have been reported, but poor compliance remains a barrier. Smartphone-based interventions may improve compliance, but data on its use in women with recent GDM are limited.

**Objective:**

This trial aimed to investigate the efficacy of a smartphone app in restoring optimal weight following delivery in women with GDM, in the setting of a population with high rates of GDM and type 2 diabetes.

**Methods:**

In this unblinded randomized controlled trial, 200 women with GDM were randomized to receive the intervention or standard care following delivery. The intervention enabled logging of weight, meals, and activity, with web-based interaction with a team comprising dieticians, a physiotherapist, and an occupational therapist. The primary outcome was an achievement of optimal weight (defined as the restoration of first trimester weight if first trimester BMI≤23 kg/m^2^ or weight loss of at least 5% from first trimester weight if first trimester BMI>23 kg/m^2^) at 4 months post partum. Secondary outcome measures included absolute weight loss, serum metabolic markers, self-reported nutritional intake, health education, and quality of life via questionnaires and user engagement in the intervention group.

**Results:**

In total, 40% (38/96) of women in the intervention group achieved optimal weight at 4 months post delivery compared with 32% (28/93) in the control group (*P*=.27). Compared with the control group, women in the intervention group reported significantly reduced caloric intake at 4 months after delivery (*P*<.001) and higher health-directed behavior scores (*P*=.045). The intervention group also reported increased emotional distress scores (*P*=.01). At 4 months, participant engagement with the intervention was maintained at 60.8% (SD 33.9%).

**Conclusions:**

Although a statistically significant increase in women achieving healthy weight was not observed, this app remains promising, as women in the intervention group reported improved health behaviors and lower caloric intake. Importantly, the high retention rates suggest that a larger study with a longer follow-up period might confirm the effectiveness of this app for weight management.

**Trial Registration:**

ClinicalTrials.gov NCT03324737; https://clinicaltrials.gov/ct2/show/NCT03324737

**International Registered Report Identifier (IRRID):**

RR2-10.1186/s12889-019-7691-3

## Introduction

### Background

Type 2 diabetes mellitus (T2DM) is an important cause of morbidity and mortality, estimated to have affected 415 million people worldwide in 2015 [1]. Women with gestational diabetes mellitus (GDM) in their pregnancies have up to seven-fold increased risk of developing subsequent T2DM [2]. The postpartum period following GDM represents a unique opportunity for early intervention to lower the risk of subsequent T2DM, possibly delaying the point at which prediabetes or T2DM is detected through health screening or other clinical encounters at later stages in their lives.

Weight gain following pregnancy has been shown to increase T2DM risk in women with a history of GDM [3]. A post hoc analysis of women with a history of GDM recruited in the diabetes prevention program showed that weight loss through intensive lifestyle modification reduced T2DM risk by 53% [4]. Although there is often ready acceptance of lifestyle changes among women during the antenatal period after the diagnosis of GDM [5], adherence to these lifestyle changes after delivery remains to be a challenge. This is especially so in the early postnatal period, where the challenges of recovering from the delivery process, breastfeeding, and caring for a newborn all lead to a chronic lack of sleep and fatigue [6,7].

Telephone-based [8] and face-to-face [9,10] interventions have been shown to be moderately effective in reducing postpartum weight retention in women with GDM, but poor compliance due to time constraints is a constantly cited barrier to successful postpartum weight loss [9,11]. In addition, these methods are labor-intensive and may depend on the busy mother being available at definite time slots. Questionnaires indicate that women with GDM in Singapore prefer web-based health education or apps for health education [12], which is consistent with findings from a trial that showed improved postpartum weight loss in women with GDM using a web-based intervention [13]. App-based interventions appear to be effective in improving nutritional behaviors and weight loss in the general population [14], but the literature on app-based interventions for weight loss during or after pregnancy is limited [15-20]. Reduced gestational weight gain has been reported with the use of an app in the antenatal period [17,19], but a positive effect on postpartum weight retention has yet to be demonstrated [18]. To date, there have been no studies on the use of apps for postpartum weight loss in women with a history of GDM.

Singapore has one of the highest rates of GDM globally, reaching up to 25% of pregnancies [21], as well as one of the highest smartphone penetration rates in Asia (approximately 90%) [22], both of which contribute to the clinical relevance and feasibility of this trial in this population.

### Objectives

The Smartphone App to Restore Optimum Weight (SPAROW) trial was a randomized controlled trial (RCT) undertaken to examine the effectiveness of an interactive app to restore optimal weight in postnatal women with a recent history of GDM. We measured the ability of the app to achieve first trimester weight in subjects at 4 months post partum if their first trimester BMI was within the normal range or weight loss of at least 5% of first trimester pregnancy weight if they were overweight. Other outcomes examined were the effects of the app on dietary and health education parameters and on a series of cardiometabolic markers. Our hypothesis was that a smartphone app would be effective in achieving optimal weight in women with GDM.

## Methods

### Study Design

The detailed design and methodology of the SPAROW trial are described elsewhere [12]. In brief, participants were recruited from women who had recently delivered at the National University Hospital (NUH), Singapore. The trial obtained ethics approval from the Domain Specific Review Board and was registered at ClinicalTrials.gov on October 30, 2017 (identifier: NCT03324737).

### Recruitment

The electronic medical records of all women in the postnatal ward were screened daily by the research members to determine eligibility. If eligibility criteria were met, women were approached by a member of the study team in the ward before discharge, and written informed consent was obtained.

### Participants

Eligible women included postpartum women aged ≥21 years diagnosed with GDM antenatally (between 24-34 weeks of gestation) using a 75-g 3 time point oral glucose tolerance test (OGTT) according to the 2013 World Health Organization criteria (fasting plasma glucose ≥5.1 mmol/L, 1-hour plasma glucose ≥10.0 mmol/L, and/or 2-hour plasma glucose ≥8.5 mmol/L) [23]. Women were required to own a smartphone and be able to use an app independently**.** In addition, the first trimester weight must have been documented at or before 13 weeks. Women with pre-existing type 1 diabetes mellitus or T2DM and women who delivered before 36 weeks were excluded.

### Randomization and Enrollment

Participants were randomized at the recruitment visit to the intervention or control arm using a random permuted block design with a block size of 4. An independent researcher generated the set of sequences and assigned participants to the intervention or control groups using sequentially numbered sealed opaque envelopes to ensure allocation concealment until the interventions were assigned. Owing to the nature of the intervention, blinding of participants and assessors was not possible**.**

### Standard Care Group (Control)

Women in the standard care arm were given a follow-up appointment at 6 weeks post partum for review by a clinician with a routine postnatal check where dietary advice was provided and a repeat OGTT was performed. Women found to have impaired fasting glucose or impaired glucose tolerance were issued a letter reinforcing healthy lifestyle changes and encouraged to consult a family physician. Participants with T2DM were referred to their family physician or internist of their choice. Women with a normal OGTT were informed of their normal results.

### App Group (Intervention)

Women allocated to the intervention arm were instructed to download an app called *Nutritionist Buddy (nBuddy)* immediately post partum before discharge and were briefed on its use by a member of the study team. During this briefing, participants were taught how to use the different functions of the app, and the live chat function was demonstrated. A colored information booklet was also provided to the participant on how to use the app. The nBuddy app is locally developed and was based on the Obesity-Related Behavioral Intervention Trials model for developing behavioral treatments for chronic disease [24]. This has been described in further detail in our protocol [12].

Through the app, participants were encouraged to work toward personalized weight targets. Calorie and activity level goals set to attain the target weight were individualized according to participants’ profiles and were automatically generated by the app. Breastfeeding status was factored into the eventual recommended caloric goal and adjusted accordingly if women stopped breastfeeding. If participants had difficulty achieving goals that were initially set, the targets were readjusted by members of the study team in agreement with the participants. Once the initial target weight was achieved, new goals were set to attain the final target weight.

Participants were advised to log their food intake daily by selecting a pre-entered meal from the app’s nutritional database of more than 11,000 locally available foods. Reminder notifications were sent 2 hours after the standard meal times if participants failed to do so. Prompts recommending healthier food alternatives catered to ethnicity were activated automatically if foods that were unhealthy or high in calories were selected or if participants had exceeded their calorie limit for the day. Optional prompts for meal planning could be selected by participants, which would suggest healthy food options 1 hour before meal time.

The step-counting function allowed users to track their physical activities. Participants were also able to manually input a range of physical activities, which were then automatically translated into step counts.

A total of 16 video clips covering themes such as diet, exercise, and emotional health for new mothers, as well as the benefits of breastfeeding and weaning diet for babies, were specially produced for the study. These clips were short (3 minute) to cater to time-challenged mothers and were made available for viewing at appropriate times after delivery [12].

A unique feature of the intervention was that it allowed live interaction between the participants and the study team consisting of dietitians, physiotherapists, and occupational therapists. All members of the research team attended a 1-hour training session conducted by the chief dietician on how to support the participants through the app. After the app was downloaded by the participant, the study team member who recruited the participant in the ward initiated the first live chat on the same day. Following this, team members actively engaged participants on the web for 5 minutes to 10 minutes daily during weekdays for the first week, followed by 2 to 3 times a week in subsequent weeks. Questions raised by participants throughout the week were responded to within a working day. Issues faced by the participants were discussed between team members to ensure the provision of appropriate and relevant advice. In addition, adjustment strategies and self-care behaviors were encouraged to aid participants in adapting to motherhood and healthy habit formation.

### Data Collection

The first trimester weight was defined as the weight taken before 13 weeks of gestation. If multiple weights were available in the first trimester, the mean of the weights was taken. Participants in the control and intervention arms were scheduled for follow-up visits at 6 weeks and 4 months postnatally, during which a series of investigations were performed [12]. A calibrated digital weighing machine (Seca 799) was used for all clinic-recorded weights, with an accuracy of 50 g for <50 kg and 100 g for 50-150 kg.

Data on app usage by participants were manually retrieved via the app’s backend dashboard in combination with usage statistics obtained from the app developer. We tracked engagement data in the following categories: (1) meal logging, (2) step logging, (3) weight logging, and (4) interactivity with health coaches. Owing to the lack of available app engagement data in the current literature, we defined adequate engagement as logging of at least one meal per day for more than 50% of the month, logging or syncing of steps at least 8 times per month, logging of weight at least 4 times per month, and communicating with coaches on the app at least 8 times per month. The overall utilization rate represented the percentage of days in a month in which an app component was engaged. To standardize calculations, it was assumed that each month had a total of 30 days.

Data were also extracted from the step count function of the app for the intervention group at baseline, 6 weeks, and 4 months.

Three-day food diaries were mailed to the participants 2 weeks before their follow-up visits. They were advised to input meals consumed on 2 weekdays and 1 weekend day and submit them at the follow-up visit.

The Health Education Impact Questionnaire (heiQ), RAND-12, and self-efficacy questionnaires were self-administered at the follow-up visits. The heiQ questionnaire is a well-validated tool used to assess self-management support and patient education programs [25] and for evaluating interventions for chronic conditions such as T2DM [26-28]. It is a self-reported questionnaire with 42 items divided into 8 subscales. Higher scores on each subscale reflect higher levels of skill, knowledge, or understanding, with the exception of the emotional distress subscale, which is reversed, and higher scores indicate higher levels of distress. The RAND-12 is a well-validated subjective measure of quality of life, developed from the Short-Form Health Survey (12 items) [29]. It is a self-administered global questionnaire with 12 questions that are grouped into 2 domains: physical and mental health component scores. The Self-Efficacy to Regulate Exercise questionnaire assesses participants’ confidence in their ability to exercise regularly in specific circumstances [30].

### Study Outcomes

The primary outcome measure of this study was the percentage of women who were able to achieve their first trimester weight at 4 months post partum if their first trimester BMI was ≤23 kg/m^2^ or weight loss of at least 5% with respect to first trimester weight if their first trimester BMI was >23 kg/m^2^.

Prespecified secondary outcomes were as follows:

1. A 75 g 2-hour OGTT, glycated hemoglobin (HbA_1c_), C-peptide, homeostatis model assessment of insulin resistance, lipid profiles, liver function, high-sensitivity C-reactive protein, and interleukin-6.
2. Mean absolute weight loss.
3. Breastfeeding status.
4. Blood pressure.
5. Right hand grip strength and waist circumference.
6. heiQ, self-efficacy, and RAND-12 questionnaire.
7. Caloric and macronutrient intake assessed by a 3-day food diary.

### Statistical Analysis and Sample Size

The sample size was estimated based on the primary outcome of attaining optimal weight at 4 months post delivery. This was defined as their first trimester weight if their first trimester BMI was ≤23 kg/m^2^ or weight loss of at least 5% if their first trimester BMI was >23 kg/m^2^. We estimated that 20% of women in the control group would attain their target weight based on similar studies [8]. An additional 20% of women attaining their target weight in the intervention group was thought to be clinically significant, corresponding to 40% of women receiving the intervention. We calculated that 75 individuals in each group would be required to achieve a power of at least 80%. Accounting for an approximate attrition rate of 15%, we aimed to recruit 100 individuals in each group.

For the analysis of the outcomes based on weight restoration and breastfeeding status (exclusive, partial, or none), the Pearson *χ*^2^ test was used to compare the respective proportions between groups at 4 months post partum. The effect estimate was expressed as an odds ratio (OR) with a 95% CI. Subsequent analyses via the mixed effect logistic regression model were used to adjust for ethnicity, parity, and the effect of time, taking into account repeated measures at 6 weeks and 4 months.

For secondary outcomes involving absolute weight change, anthropometric measurements, diet and nutrition markers as well as RAND-12, self-efficacy, and heiQ domains at 4 months post partum, the mean difference between the intervention and control groups was first compared based on a two tailed *t* test. Furthermore, adjusted analyses were performed using the linear mixed model to adjust for the respective baseline covariate (where available) and the effect of time, taking into account possible intrasubject correlation between the repeated outcomes at 6 weeks and 4 months.

All analyses were performed on an intention-to-treat (ITT) basis, assuming a two-sided test at the 5% level of significance using StataCorp 2019 (StataCorp LLC).

## Results

### Study Population

**Figure 1:**
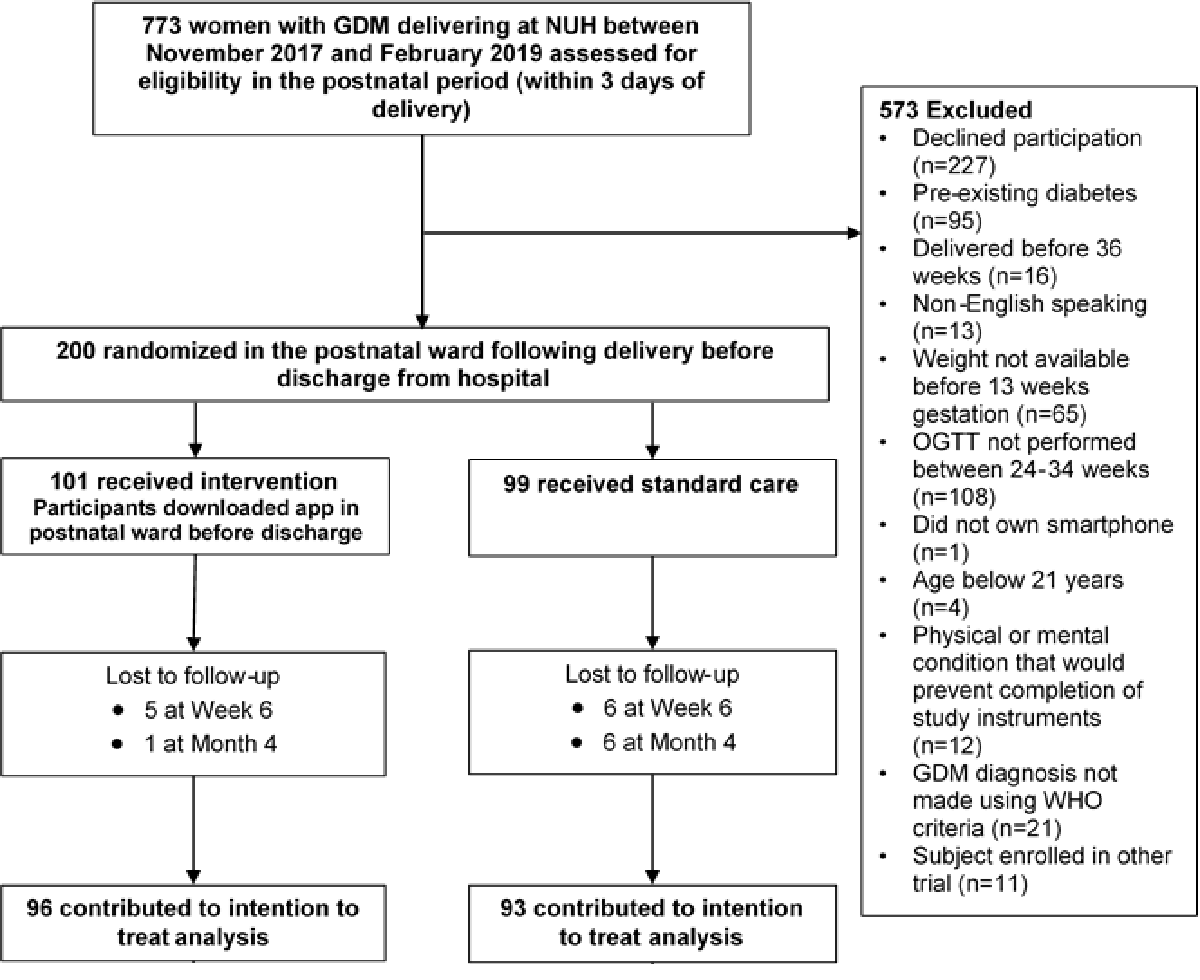
Trial flow. GDM: gestational diabetes mellitus; NUH: National University Hospital; OGTT: oral glucose tolerance test; WHO: World Health Organization.

Women with GDM (n=773) who delivered at NUH, Singapore, between November 2017 and February 2019 were screened for eligibility (Figure 1). A total of 346 women did not meet the eligibility criteria, and 227 declined participation. In total, 200 women consented to participate in the trial and were randomized either to the intervention arm (n=101) or the control arm (n=99). At the end of the study, 95% of those recruited (96 in intervention and 93 in control) provided information on the outcome analyses for the duration that they were observed. In total, 11 patients (5 intervention and 6 control) were lost to follow-up at week 6 and a further 7 (1 intervention and 6 control) were lost to follow-up at month 4.

The characteristics of the participants who contributed to the outcome analyses are shown in Table 1. The baseline characteristics were largely comparable between the control and intervention groups. The mean gestational weight gain did not differ significantly between the groups.

**Table 1:** Demographics and clinical characteristics of trial participants.

Characteristics		Intervention (n=96)	Control (n=93)	Total (N=189)
Age (years), mean (SD)		32.6 (4.5)	32.4 (4.2)	32.5 (4.3)
**Ethnicity, n (%)**				
	Chinese	46 (47.9)	54 (58.1)	100 (52.9)
	Malay	35 (36.5)	22 (23.7)	57 (30.2)
	Indian	11 (11.5)	13 (14.0)	24 (12.7)
	Others	4 (4.2)	4 (4.3)	8 (4.2)
Height at delivery (m), mean (SD)		1.58 (0.06)	1.60 (0.05)	1.59 (0.06)
First trimester weight (kg), mean (SD)		63.1 (16.0)	61.9 (11.3)	62.5 (13.9)
First trimester BMI (kg/m^2^), median (IQR)		24.6 (20.7-27.8)	23.4 (21.0-27.0)	23.7 (20.7-27.6)
BMI at delivery (kg/m^2^), median (IQR)		26.9 (23.0-29.5)	25.8 (22.8-28.6)	26.2 (22.9-28.9)
**Blood pressure at recruitment (mm Hg), mean (SD)**				
	Systolic	107.1 (11.6)	106.8 (10.7)	106.9 (11.2)
	Diastolic	63.7 (9.3)	63.3 (9.9)	63.5 (9.6)
**Parity, n (%)**				
	1	39 (40.6)	48 (51.6)	87 (46.0)
	2	38 (39.6)	33 (35.5)	71 (37.6)
	3+	19 (19.8)	12 (12.9)	31 (16.4)
**Level of education, n (%)**				
	Secondary	11 (11.5)	6 (6.5)	17 (9.0)
	GCE^a^ A level or IB^b^ or ITE^c^ or NTC^d^	5 (5.2)	6 (6.5)	11 (5.8)
	Diploma or advanced diploma or polytechnic	21 (21.9)	19 (20.4)	40 (21.2)
	Bachelor’s degree	49 (51.0)	40 (43.0)	89 (47.1)
	Postgraduate	10 (10.4)	22 (23.7)	32 (16.9)
Employed, n (%)		74 (77.1)	80 (86.0)	154 (81.5)
History of previous cesarean section, n (%)		22 (23.7)	20 (22.5)	42 (23.1)
Pre-existing hypertension, n (%)		1 (1.0)	1 (1.1)	2 (1.1)
History of gestational diabetes in previous pregnancies, n (%)		13 (13.5)	13 (14.0)	26 (13.8)
History of impaired fasting glucose or glucose tolerance, n (%)		0 (0.0)	1 (1.1)	1 (0.5)
**Oral glucose tolerance test at diagnosis (mmol/L), mean (SD)**				
	Fasting	4.6 (0.4)	4.7 (0.5)	4.6 (0.5)
	1 hour	9.8 (1.4)	10.2 (1.4)	10.0 (1.4)
	2 hours	8.4 (1.4)	8.7 (1.2)	8.5 (1.3)
Gestational age at gestational diabetes diagnosis (weeks), mean (SD)		27.1 (2.0)	27.0 (1.7)	27.1 (1.8)
HbA_1c_^e^ at diagnosis (%), mean (SD)^f^		5.4 (0.3)	5.2 (0.4)	5.3 (0.4)
Gestational weight gain (kg), mean (SD)		8.84 (4.2)	9.19 (4.7)	9.0 (4.5)

^a^GCE: General Certificate of Education.

^b^IB: International Baccalaureate.

^c^ITE: Institute for Technical Education.

^d^NTC: National Technical Certificate.

^e^HbA_1c_: glycated hemoglobin.

^f^12 observations with missing HbA_1c_ measurements at diagnosis.

### Primary Outcome

In the ITT analysis, 40% (38/96) of women in the intervention arm achieved optimal weight at 4 months compared with 30% (28/93) in the control arm (OR 1.40, 95% CI 0.76 to 2.58). Adjustment for ethnicity, parity, and the effect of time via the linear mixed model did not materially alter the results (OR 1.55, 95% CI 0.53 to 4.54).

### Secondary Outcomes

Although women in the intervention arm achieved a mean difference in weight reduction of 1.05 kg compared with the control arm (95% CI 0.14 to 2.24; *P*=.08), this difference was not statistically significant. There were no significant differences in anthropometric measurements, breastfeeding status, and OGTT results at 4 months (Table 2). Metabolic serum markers did not differ between the groups at 4 months, except for lower HbA_1c_ levels in the control group at 4 months (Multimedia Appendix 1). At 4 months postnatally, 3% (3/87) and 0% (0/95) had impaired fasting glucose and 14% (12/87) and 19% (18/95) had impaired glucose tolerance in the intervention and control groups, respectively. None of the participants had developed T2DM at 6 weeks or 4 months.

**Table 2:** Mean anthropometric measurements in the intervention and control group and their mean differences at month 4^a^.

Outcome		Intervention (n=96)	Control (n=93)	Mean difference (95% CI)	*P* value
**Absolute difference in weight (kg) from first trimester**					
	Unadjusted	−1.39	−0.34	−1.05 (−2.24 to 0.14)	.08
	Adjusted	−0.63	0.08	−0.71 (−1.76 to 0.33)	.18
**Waist circumference (cm)**					
	Unadjusted	83.7	85.0	−1.26 (−4.41 to 1.89)	.43
	Adjusted	84.1	85.6	−1.46 (−4.42 to 1.52)	.34
**Systolic blood pressure (mm Hg)**					
	Unadjusted	106.8	106.7	0.06 (−3.09 to 3.22)	.97
	Adjusted	107.0	106.7	0.23 (−2.41 to 2.88)	.86
**Right hand grip strength (kg)**					
	Unadjusted	22.46	23.36	−0.90 (−2.30 to 0.50)	.21
	Adjusted	22.40	22.84	−0.44 (−1.77 to 0.89)	.52
**Exclusive breastfeeding (days)**					
	Unadjusted	51.6	52.9	0.95 (0.53 to 1.70)	.86
	Adjusted	50.5	48.3	1.26 (0.41 to 3.86)	.68
**Fasting OGTT^b^ test (mmol/L)**					
	Unadjusted	4.77	4.75	0.02 (−0.12 to 0.16)	.76
	Adjusted	4.73	4.74	−0.02 (−0.12 to 0.08)	.73
**2-hour OGTT (mmol/L)**					
	Unadjusted	6.59	6.65	−0.06 (−0.56 to 0.43)	.80
	Adjusted	6.63	6.58	0.05 (−0.39 to 0.48)	.83

^a^Adjusted analysis is based on a linear mixed effect model adjusting for the respective baseline covariate (in addition, ethnicity and parity were also included for exclusive breastfeeding) and the effect of time, taking into account possible intrasubject correlation between the repeated outcomes at week 6 and month 4.

^b^OGTT: oral glucose tolerance test.

### Diet and Nutrition

At 6 weeks, women in the intervention group reported reduced total caloric intake (−591.3 kcal, 95% CI −717.1 to −465.4), total fat (−25.3 g, 95% CI −31.7 to −19.0), protein (−24.1 g, 95% CI −31.1 to −17.0), carbohydrate (−67.4 g, 95% CI −84.6 to −50.2), and sugar (−22.9 g, 95% CI −30.0 to −15.7) compared with controls. Similarly, at 4 months, the intervention group reported reduced total caloric intake (−614.2 kcal, 95% CI −751.5 to −476.9), total fat (−20.7 g, 95% CI −27.2 to −14.2), protein (−25.7 g, 95% CI −33.1 to −18.3), carbohydrate (−71.2 g, 95% CI −90.3 to −52.1), and sugar (−27.9 g, 95% CI −35.7 to −20.1; Table 3). The results remained similar even after adjusting for baseline covariates and the effect of time. The relative contribution of sugar to overall caloric intake was significantly lower in the intervention arm, with an adjusted mean difference of −0.019 (95% CI −0.028 to −0.010; *P*<.001; Table S2 in Multimedia Appendix 1). The relative contributions of fat, protein, and carbohydrate did not differ significantly between the groups, suggesting that a reduction in sugar intake was the likely driver for reduced overall caloric intake in the intervention arm.

**Table 3:** Mean nutritional intake in the intervention and control and their mean differences at month 4^a^.

Diet and nutrition markers		Intervention (n=95)^b^	Control (n=87)^b^	Mean difference (95% CI)	*P* value
**Calories (kcal)**					
	Unadjusted	1441.3	2055.5	−614.2 (−753.3 to −475.2)	<.001
	Adjusted	1463.2	2065.9	−602.7 (−717.6 to −487.9)	<.001
**Total fat (g)**					
	Unadjusted	70.7	91.4	−20.7 (−27.4 to −14.2)	<.001
	Adjusted	64.0	87.0	−23.0 (−28.3 to −17.8)	<.001
**Protein (g)**					
	Unadjusted	56.1	81.8	−25.7 (−33.1 to −18.3)	<.001
	Adjusted	64.4	89.3	−24.9 (−30.8 to −19.0)	<.001
**Carbohydrate (g)**					
	Unadjusted	168.5	239.7	−71.2 (−90.3 to −52.1)	<.001
	Adjusted	169.2	238.5	−69.3 (−84.9 to −53.7)	<.001
**Sugar (g)**					
	Unadjusted	37.8	65.7	−27.9 (−35.7 to −20.1)	<.001
	Adjusted	38.6	64.0	−25.4 (−31.3 to −19.5)	<.001

^a^Adjusted analysis based on a linear mixed effect model adjusting for the respective baseline covariate and the effect of time, taking into account possible intrasubject correlation between repeated outcomes at week 6 and month 4.

^b^In addition to the 11 women who were lost to follow-up at week 6, diet and nutrition markers were not available at week 6 for 7 women (1 intervention and 6 control).

### Physical Activity

Participants in the intervention group logged a mean step count of 4065 (95% CI 3483.6 to 4645.7) at 6 weeks. The mean step count increased to 4880 (95% CI 4195.4 to 5565.5) at 4 months (*P*=.04).

### Health Behaviors

Women in the intervention group reported higher health-directed behavior scores on the heiQ questionnaire (mean difference 0.16, 95% CI 0.004 to 0.32) than controls at 4 months (Table S3 in Multimedia Appendix 1). The health-directed behavior component of the heiQ questionnaire assesses changes in lifestyle through healthy behaviors such as diet, exercise, and relaxation routines aimed at either disease prevention and/or health promotion. High scores indicated high levels of healthy behaviors [25]. Specific questions that comprise the health-directed behavior component of the heiQ are presented in Table 4.

**Table 4:** Percentage of “agree” and “strongly agree” responses to questions in the health-directed behavior component of the Health Education Impact Questionnaire in the intervention group.

Question	Week 6, n (%)	Month 4, n (%)
On most days of the week, I do at least one activity to improve my health (eg, walking, relaxation, exercise)	85 (84.2)	95 (90.5)
I do at least one type of physical activity every day for at least 30 min (eg, walking, gardening, housework, golf, bowls, dancing, Tai Chi, swimming)	75 (74.3)	95 (80.0)
On most days of the week, I set aside time for healthy activities (eg, walking, relaxation, exercise)	67 (67.7)	65 (68.4)
I walk for exercise, for at least 15 min per day, most days of the week	83 (82.2)	82 (86.3)

However, increased emotional distress scores were observed in the intervention group (0.21, 95% CI 0.05 to 0.38). Women in the intervention group also reported lower scores on the physical component summary of the RAND-12 questionnaire (*P*=.04), but this difference was no longer statistically significant after adjustment (Table S3 in Multimedia Appendix 1).

At 4 months, women in the intervention group reported higher scores in question 6 (*P*=.02, unadjusted value) and 11 (*P*=.04, unadjusted value) of self-efficacy to regulate the exercise questionnaire. This addressed how confident participants felt in being able to perform their exercise routine regularly (3 or more times a week) despite physical discomfort experienced during exercise (question 6) and when they had other time commitments (question 11; Table S4 in Multimedia Appendix 1).

### User Engagement

The participants’ engagement with the app is presented in Table 5. On average, the usage of at least one component of the app by participants was 70.8% in month 1 and 60.8% in month 4. In the first month, 62.4% (63/101), 83.2% (84/101), and 78.2% (79/101) of users logged their meals, step counts, and weight (as defined earlier), which dropped to 41.6%, 76%, and 61.4% at month 4, respectively.

**Table 5:** Participant engagement with the app in the intervention group.

App usage by participants (n=101)	Month 1	Month 2	Month 3	Month 4	4-month average
Overall utilization rate (Percentage of days an app component was used per user), mean (SD)	70.8 (30.6)	69.3 (30.9)	61.3 (34.0)	60.8 (33.9)	65.5 (29.0)
Percentage of users who logged meals (≥15 times a month), n (%)	63 (62.4)	63 (62.4)	50 (49.)5	42 (41.6)	54 (54.0)
Percentage of users with step count log (≥8 times a month), n (%)	84 (83.2)	85 (84.2)	78 (77.2)	77 (76.2)	81 (80.2)
Percentage of users with weight log (≥4 times a month), n (%)	79 (78.2)	71 (70.3)	60 (60.4)	62 (61.4)	68 (67.6)
Percentage of users with interactive exchange (≥8 times a month), n (%)	33 (32.7)	31 (30.7)	21 (20.8)	20 (19.8)	26 (25.7)

## Discussion

### Overview of the Findings

In this RCT involving an app-based lifestyle intervention among postpartum women with recent GDM, the intervention did not achieve a statistically significant difference in the primary outcome. However, the intervention was effective in promoting an overall healthier lifestyle with reduced self-reported caloric intake and improved health-directed behaviors in women with a history of GDM. Utilization of the app remained to be constantly high throughout the study period, with 60% usage of the intervention at 4 months.

The average utilization rate in postnatal women appears to be comparable with populations utilizing apps for chronic disease management, which reported nonengagement rates as high as 40% in a recent review [31]. Across postnatal lifestyle interventions, the engagement level in this app-based study was significantly higher than in studies relying on telephone-based and face-to-face interventions, which reported full attendance rates of only 10% to 15% [8,9]. Convenience and portability may be key factors associated with greater app engagement in time-pressed postnatal women, which in turn may lead to better recommendation adherence and outcomes [32]. A pilot study investigating the use of a smartphone intervention for postpartum weight loss in 40 women who were mostly overweight or obese showed no significant improvement in weight loss at 16 weeks. However, post hoc analysis showed a significant difference in women who were compliant (>70% adherence) to the intervention, suggesting a possible benefit with increased compliance [18]. Our study provides support for this easily scalable app intervention to be applied to larger numbers of subjects to assess its ability to achieve significant weight loss.

A possible reason for our trial being unable to detect a clear improvement in achievement of optimal weight, the primary outcome, was likely due to a higher proportion of women achieving optimal weight in the control arm of 32.2% than the expected 20% based on other studies [8]—the postulated proportion that was used to estimate the sample size for this trial. The continued application of dietary advice obtained from regular dietitian reviews in the antenatal period could have contributed to the higher than expected rates of weight loss for women in the control arm. Another possible explanation for this difference is the Hawthorne effect, wherein women in the control arm might be more likely to modify their health behaviors in response to their awareness of being observed, resulting in social desirability bias. In addition, women who agreed to participate in the study were also likely to represent a highly motivated group of individuals compared with the general population, which could explain the higher rate of optimal weight achievement in the control arm.

The ethnic makeup in the 2 arms differed slightly, with fewer Chinese and more Malays in the intervention arm. Although Malay and Indian people are known to have greater metabolic risk and adiposity [33], adjustment for ethnicity did not alter the results substantially; therefore, ethnicity is unlikely to be a major contributor to our results.

Women who received the intervention reported significantly reduced caloric intake by over 600 calories, which was mainly due to a reduction in sugar intake in the intervention group. Although this finding needs to be interpreted with caution because of inherent limitations of self-reported food diary studies, our findings are consistent with a meta-analysis of data from 41 studies by Villinger et al [14] showing that app-based mobile interventions are effective for changing nutrition behaviors in a wide range of study settings.

Women who received the intervention reported improved health-related behaviors, as evidenced by the statistically significant increase in scores in the health-related behavior domain of the heiQ questionnaire. Similarly, a study investigating the use of a mobile health intervention among people with T2DM showed higher scores in the skill and technique acquisition domain of the heiQ questionnaire after 1 year [34]. Women in the intervention also reported significantly higher scores in 2 questions of the self-efficacy to exercise questionnaire, which assessed their ability to persist with regular exercise despite physical discomfort and time constraints. This suggests that the intervention may improve health behaviors, which could possibly translate into significant weight loss over a longer period of time.

Increased step counts between week 6 and month 4 were noted in the intervention group. The overall step counts, although increased, were modest compared with those in other studies, which achieved up to 10,000 steps per day [35]. However, step counts were obtained from the step count function of the smartphone rather than a pedometer, and hence may be an underrepresentation of the actual number of steps taken.

The intervention was unique in that it had a large databank of popular Southeast Asian street foods and had built-in prompts to suggest healthier alternatives that were culturally appropriate, an important feature in Singapore’s multiethnic society. Its interactive features enabled real-time inputs to inculcate adjustment strategies and self-care behaviors, as well as specially commissioned videos relevant to the recently parturient mother in Asian societies.

Women receiving the intervention reported higher emotional distress scores than those receiving the intervention. Lower scores in the physical component summary but not the mental component summary of the RAND-12 questionnaire in the intervention group may suggest that the source of distress was related to physical fitness rather than emotional problems. These findings may be explained by an increased awareness of potentially developing T2DM and the demands of adhering to lifestyle and dietary modifications to reduce this risk—all of which may increase emotional distress in women receiving the intervention in the short term. These findings differ from trials on the use of an app in the antenatal period to reduce gestational weight gain, which showed no change in mood and quality of life with the intervention [36]. However, the study also showed that women reported higher depression and lower physical quality of life scores during pregnancy than during the postpartum period. This may reflect the different sources of background stressors during the different stages of the pregnancy journey, which may moderate the interaction between the intervention and quality of life.

Although there are several trials investigating the use of lifestyle interventions to improve postpartum weight loss in women with and without GDM [8,13,37,38], this is the first RCT investigating the use of an app for postpartum weight loss in women with recent GDM. Our trial demonstrated that an app supported by interactions with a group of health care professionals may improve health behaviors in postpartum women and sustain high user engagement. This resulted in reduced caloric intake and improved health behaviors when assessed by self-report. As such apps for diet and nutritional intake tracking are currently available globally, implementation of similar studies in other centers would be feasible. In addition, our study population likely represents the ethnic groups of many Southeast Asian countries in which women are known to have a high prevalence of GDM and T2DM.

### Limitations

The main limitation of our study was that it was underpowered to detect a statistically significant difference in the primary outcome, likely due to the reasons mentioned earlier. Another limitation is that the outcomes in which significant improvements were detected (caloric-intake and health-directed behaviors) were self-reported rather than objectively measured outcomes. Studies have shown that self-reported physical activity is often an overestimation of actual physical activity [39], which could also explain the lack of significant improvement in weight loss in the intervention group. This emphasizes the need for objectively measured physical activity in future studies, possibly in the form of pedometers provided to all participants in the study. We also acknowledge the limitations of interpreting decreased caloric intake without concomitant data on energy expenditure, as reduced caloric intake may be related to reduced physical activity during the postnatal period. However, as changes in body weight and breastfeeding may be on the causal pathway between the intervention and caloric intake, it is not appropriate to adjust for these factors in our regression model. Similarly, controlling for overall caloric intake when comparing differences in specific macronutrients may not be appropriate, as overall caloric intake lies in the causal pathway between intervention and macronutrient intake. A separate modeling study to investigate the causal pathways involved will be conducted in the future.

The challenge with any intervention remains its ability to sustain long-term maintenance of optimal weight, as positive effects on weight loss have been shown to be negated following the cessation of the intervention [8]. However, there is some evidence to suggest that long-term follow-up following an intervention may lead to sustained weight loss up to 13 years postintervention [40]. This implies that if women continue with the intervention for long enough, healthier food and lifestyle choices may become routine, which may be key in sustaining optimal weight and reducing T2DM risk in these women. Given the apparent acceptability of this app intervention with relatively high and sustained engagement rates, it is possible that long-term follow-up of these women would demonstrate even greater weight loss or weight maintenance, which is a crucial step in the battle against the growing T2DM epidemic. In addition, app-based interventions would be particularly valuable in the delivery of care during the current COVID-19 pandemic and its aftermath, which will rely heavily on telemedicine to minimize direct patient contact.

### Conclusions

This RCT suggests that an app may improve health behaviors and can maintain high user engagement. The results of this study support the need for a larger RCT with greater statistical power and longer-term follow-up to track long-term weight loss and cardiometabolic outcomes.

## Appendix

Multimedia Appendix 1 Supplementary Tables.

Multimedia Appendix 2 CONSORT-eHEALTH checklist (V1.6.1).

## Abbreviations

GDM: gestational diabetes mellitus
HbA_1c_: glycated hemoglobin
heiQ: Health Education Impact Questionnaire
ITT: intention-to-treat
NUH: National University Hospital
OGTT: oral glucose tolerance test
OR: odds ratio
RCT: randomized controlled trial
SPAROW: The Smartphone App to Restore Optimum Weight
T2DM: type 2 diabetes mellitus
