# Supplementary material for: A Smartphone App to Restore Optimal Weight (SPAROW) in Women With Recent Gestational Diabetes Mellitus: Randomized Controlled Trial
Source: JMIR Mhealth Uhealth. 2021 Mar 16;9(3):e22147. doi: 10.2196/22147 (PMC8088857; doi:10.2196/22147)
Supplement: Multimedia Appendix 1 [file mhealth_v9i3e22147_app1.docx]

**Supplementary Table 1**: Mean difference in metabolic serum makers between Intervention and Control at Month 4

| **Metabolic serum markers** | **Intervention**  **(n = 96)** | **Control**  **(n = 93)** | **Mean Difference**  **(95% CI)** | ***p*-value** |
| --- | --- | --- | --- | --- |
| **HbA1c (%)**  Unadjusted  Adjusted  **C-peptide (pmol/L)***  Unadjusted  Adjusted  **HOMA-IR***  Unadjusted  Adjusted  **Total cholesterol (mmol/L)**  Unadjusted  Adjusted  **HDL (mmol/L)**  Unadjusted  Adjusted  **LDL (mmol/L)**  Unadjusted  Adjusted  **ALT (units/L)***  Unadjusted  Adjusted  **AST (units/L)***  Unadjusted  Adjusted  **hsCRP (mg/L)***  Unadjusted  Adjusted  **Interleukin-6 (pg/mL)***  Unadjusted  Adjusted | 5.36  5.31  383.3  383.3  1.11  1.01  4.98  5.17  1.60  1.61  3.00  3.15  19.03  19.92  20.88  21.64  1.92  2.29  3.49  1.28 | 5.21  5.26  400.7  396.6  1.13  1.06  4.97  5.22  1.60  1.61  2.93  3.15  20.40  21.55  21.65  22.36  1.57  1.92  3.67  1.20 | 0.15 (0.03 to 0.26)  0.05 (-0.02 to 0.13)  0.96 (0.82 to 1.12)  0.97 (0.84 to 1.12)  0.98 (0.78 to 1.23)  0.95 (0.78 to 1.17)   - 1. (-0.25 to 0.28)   -0.05 (-0.31 to 0.20)  0.003 (-0.11 to 0.12)  -0.001 (-0.11 to 0.11)  0.06 (-0.16 to 0.29)  0.001 (-0.23 to 0.23)  0.93 (0.81 to 1.07)  0.92 (0.81 to 1.05)  0.96 (0.89 to 1.04)  0.97 (0.90 to 1.04)  1.22 (0.86 to 1.74)  1.19 (0.88 to 1.62)  0.95 (0.73 to 1.24)  1.08 (0.88 to 1.33) | 0.013  0.180  0.574  0.642  0.854  0.660  0.915  0.686  0.958  0.991  0.588  0.996  0.324  0.227  0.355  0.362  0.267  0.251  0.716  0.446 |

**Supplementary Table 2:** Contribution of macronutrients^a^ to total caloric intake at Month 4.

| **Diet and nutrition markers** | **Intervention**  **(n = 95)** | **Control**  **(n = 87)** | **Mean Difference (95% CI)** | ***p*-value** |
| --- | --- | --- | --- | --- |
| **Fat**  Unadjusted  Adjusted  **Protein**  Unadjusted  Adjusted  **Carbohydrate (grams)**  Unadjusted  Adjusted  **Sugar (grams)**  Unadjusted  Adjusted | 0.441  0.393  0.148  0.173  0.470  0.464  0.102  0.103 | 0.406  0.381  0.159  0.179  0.465  0.461  0.125  0.122 | 0.036 (0.015 to 0.056)  0.012 (-0.001 to 0.025)  -0.011 (-0.018 to -0.004)  -0.006 (-0.019 to 0.008)  0.005 (-0.015 to 0.025)  0.002 (-0.014 to 0.019)  -0.023 (-0.035 to -0.011)  -0.019 (-0.028 to -0.010) | 0.001  0.063  0.002  0.405  0.614  0.808  < 0.001  < 0001 |

^a^ Data was calculated as follows: Fat (g) X 9/total caloric intake, Protein (g) X 4/total caloric intake, Carbohydrate (g) X 4/total caloric intake, Sugar (g) X 4/total caloric intake.

Adjusted analysis based on linear mixed effect model adjusting for the effect of time, taking into account possible intra-subject correlation between repeated outcomes at Week 6 and Month 4.

**Supplementary Table 3:** RAND12 and HeiQ Questionnaire results

| **RAND12 and HEiQ domain** | **Intervention**  **(n = 96)** | **Control**  **(n = 93)** | **Mean Difference**  **(95% CI)** | ***p*-value** |
| --- | --- | --- | --- | --- |
| **HEiQ**  **Health directed behaviour**  Unadjusted  Adjusted  **Engagement in life**  Unadjusted  Adjusted  **Self-monitoring and insight**  Unadjusted  Adjusted  **Constructive attitudes and approach**  Unadjusted  Adjusted  **Skills and technique acquisition**  Unadjusted  Adjusted  **Social integration and support**  Unadjusted  Adjusted  **Health services navigation**  Unadjusted  Adjusted  **Emotional distress**  Unadjusted  Adjusted | 3.05  2.87  3.17  3.09  3.14  3.16  3.20  3.20  3.02  3.06  3.18  3.16  3.18  3.18  2.05  1.86 | 2.89  2.72  3.08  3.07  3.10  3.17  3.16  3.16  3.00  3.04  3.10  3.14  3.11  3.16  1.84  1.70 | 0.16 (0.004 to 0.32)  0.15 (0.01 to 0.29)  0.09 (-0.02 to 0.20)  0.03 (-0.08 to 0.13)  0.04 (-0.07 to 0.14)  -0.01 (-0.10 to 0.09)  0.04 (-0.11 to 0.18)  0.04 (-0.08 to 0.15)  0.01 (-0.11 to 0.14)  0.01 (-0.08 to 0.10)  0.08 (-0.07 to 0.23)  0.02 (-0.10 to 0.14)  0.07 (-0.06 to 0.20)  0.03 (-0.07 to 0.12)  0.21 (0.05 to 0.38)  0.16 (0.03 to 0.29) | 0.045  0.036  0.096  0.618  0.481  0.886  0.592  0.534  0.839  0.806  0.309  0.780  0.313  0.618  0.010  0.018 |
| **RAND12**  **Physical Component Summary**  Unadjusted  Adjusted  **Mental Component Summary**  Unadjusted  Adjusted | 48.50  48.33  49.79  49.16 | 50.01  48.93  49.51  48.94 | -1.51 (-2.91 to -0.10)  -0.60 (-1.76 to 0.56)  0.28 (-1.54 to 2.10)  0.22 (-1.23 to 1.67) | 0.036  0.312  0.764  0.768 |

Note: Adjusted analysis based on linear mixed effect model adjusting for the effect of time, and taking into account possible intra-subject correlation between repeated outcomes at Week 6 and Month 4.

Supplementary Table 4: Difference in self-efficacy exercise domains and items between intervention and control

| **Self-efficacy to regulate exercise score**  **Exercise mean**  ***6 weeks***  Unadjusted  ***4 months***  Unadjusted  Mixed model  **Exercise emotional**  ***6 weeks***  Unadjusted  ***4 months***  Unadjusted  Mixed model  **Exercise physical state**  ***6 weeks***  Unadjusted  ***4 months***  Unadjusted  Mixed model  **Exercise motivation**  ***6 weeks***  Unadjusted  ***4 months***  Unadjusted  Mixed model  **Exercise 1**  ***6 weeks***  Unadjusted  ***4 months***  Unadjusted  Mixed model  **Exercise 2**  ***6 weeks***  Unadjusted  ***4 months***  Unadjusted  Mixed model  **Exercise 3**  ***6 weeks***  Unadjusted  ***4 months***  Unadjusted  Mixed model  **Exercise 4**  ***6 weeks***  Unadjusted  ***4 months***  Unadjusted  Mixed model  **Exercise 5**  ***6 weeks***  Unadjusted  ***4 months***  Unadjusted  Mixed model  **Exercise 6**  ***6 weeks***  Unadjusted  ***4 months***  Unadjusted  Mixed model  **Exercise 7**  ***6 weeks***  Unadjusted  ***4 months***  Unadjusted  Mixed model  **Exercise 8**  ***6 weeks***  Unadjusted  ***4 months***  Unadjusted  Mixed model  **Exercise 9**  ***6 weeks***  Unadjusted  ***4 months***  Unadjusted  Mixed model  **Exercise 10**  ***6 weeks***  Unadjusted  ***4 months***  Unadjusted  Mixed model  **Exercise 11**  ***6 weeks***  Unadjusted  ***4 months***  Unadjusted  Mixed model | 41.64  43.90  54.69  43.21  44.67  57.10  31.06  36.42  47.72  48.63  48.82  66.61  34.57  45.92  35.19  43.70  47.74  45.92  44.32  45.05  44.72  42.32  41.47  42.07  42.66  44.84  43.93  29.57  38.51  34.12  29.04  35.05  32.08  46.04  48.39  47.28  57.87  56.84  57.43  52.53  49.26  51.01  37.37  40.32  38.95 | 40.02  41.97  53.00  42.84  44.22  54.96  26.92  32.99  46.31  46.53  46.31  64.71  29.68  45.03  32.00  43.96  45.81  45.03  45.65  46.51  46.22  38.46  39.65  49.21  43.59  44.77  44.17  26.13  30.57  28.38  25.22  33.79  29.45  44.62  48.62  46.59  58.04  57.91  57.99  49.89  44.94  47.47  33.76  33.68  33.85 | 1.63 (-3.10 to 6.36)  1.93 (-2.94 to 6.79)  1.69 (-2.19 to 5.57)  0.38 (-6.08 to 6.83)  0.45 (-5.61 to 6.51)  2.14 (-3.05 to 7.34)  4.14 (-1.41 to 9.71)  3.43 (-2.33 to 9.20)  1.42 (-3.02 to 5.85)  2.10 (-3.40 to 7.60)  2.51 (-2.71 to 7.34)  1.90 (-2.56 to 6.36)  4.90 (-1.86 to 11.65)  0.88 (-5.95 to 7.71)  3.18 (-2.14 to 8.51)  -0.26 (-7.83 to 7.31)  1.93 (-5.31 to 9.17)  0.89 (-4.95 to 6.73)  -1.34 (-9.04 to 6.37)  -1.46 (-8.37 to 5.45)  -1.50 (-7.48 to 4.48)  3.85 (-3.97 to 11.68)  1.82 (-5.52 to 9.17)  2.86 (-3.49 to 9.22)  -0.93 (-8.47 to 6.61)  0.07 (-6.81 to 6.96)  -0.25 (-6.11 to 5.62)  3.45 (-3.26 to 10.15)  7.94 (1.06 to 14.81)  5.74 (0.14 to 11.34)  3.83 (-3.41 to 11.06)  1.26 (-5.45 to 7.97)  2.63 (-2.89 to 8.15)  1.42 (-5.51 to 8.35)  -0.23 (-7.02 to 6.55)  0.69 (-4.89 to 6.27)  -0.17 (-7.32 to 6.98)  -1.06 (-7.78 to 5.65)  -0.56 (-6.15 to 5.02)  2.64 (-5.70 to 10.97)  4.32 (-2.89 to 11.53)  3.54 (-2.76 to 9.85)  3.60 (-2.91 to 10.12)  6.64 (0.18 to 13.09)  5.11 (-0.09 to 10.30) | 0.498  0.435  0.393  0.909  0.883  0.419  0.143  0.242  0.532  0.452  0.344  0.404  0.154  0.801  0.242  0.946  0.600  0.766  0.733  0.678  0.623  0.332  0.625  0.377  0.809  0.983  0.934  0. 312  0.024  0.045  0.298  0.711  0.350  0.687  0.946  0.807  0.962  0.755  0.843  0.534  0.238  0.271  0.276  0.044  0.054 |
| --- | --- | --- | --- | --- |
